# Characterization of *Neisseria gonorrhoeae* isolates detected in Switzerland (1998–2012): emergence of multidrug-resistant clones less susceptible to cephalosporins

**DOI:** 10.1186/1471-2334-14-106

**Published:** 2014-02-25

**Authors:** Andrea Endimiani, Yuvia N Guilarte, Regula Tinguely, Lea Hirzberger, Sylvia Selvini, Agnese Lupo, Christoph Hauser, Hansjakob Furrer

**Affiliations:** 1 Institute for Infectious Diseases, University of Bern, Bern, Switzerland; 2 Department of Infectious Diseases, Bern University Hospital and University of Bern, Bern, Switzerland

**Keywords:** Gonococcus, ST1407, MIC, Ceftriaxone, Cefixime, MDR, NG-MAST, PBP1, PBP2, Mosaic

## Abstract

**Background:**

The spread of *Neisseria gonorrhoeae* (*Ng*) isolates resistant to the clinically implemented antibiotics is challenging the efficacy of treatments. Unfortunately, phenotypic and molecular data regarding *Ng* detected in Switzerland are scarce.

**Methods:**

We compared the characteristics of *Ng* detected during 1998–2001 (n = 26) to those detected during 2009–2012 (n = 34). MICs were obtained with the Etest and interpreted as non-susceptible (non-S) according to EUCAST criteria. Sequence type (ST) was achieved implementing the NG-MAST. *Bla*_TEM_, *ponA*, *penA*, *mtrR*, *penB*, *tet*(M), *gyrA*, *parC*, *mefA*, *ermA/B/C/F*, *rplD*, *rplV*, and 23S rRNA genes were analyzed.

**Results:**

The following susceptibility results were obtained (period: % of non-S, MIC_90_ in mg/L): penicillin (1998–2001: 42.3%, 3; 2009–2012: 85.3%, 16), cefixime (1998–2001: 0%, ≤0.016; 2009–2012: 8.8%, 0.125), ceftriaxone (1998–2001: 0%, 0.004; 2009–2012: 0%, 0.047), ciprofloxacin (1998–2001: 7.7%, 0.006; 2009–2012: 73.5%, ≥32), azithromycin (1998–2001: 11.5%, 0.25; 2009–2012: 23.6%, 0.38), tetracycline (1998–2001: 65.4%, 12; 2009–2012: 88.2%, 24), spectinomycin (1998–2001: 0%, 12; 2009–2012: 0%, 8). The prevalence of multidrug-resistant (MDR) isolates increased from 7.7% in 1998–2001 to 70.6% in 2009–2012. International STs and genogroups (G) emerged during 2009–2012 (G1407, 29.4%; G2992, 11.7%; G225, 8.8%). These isolates possessed distinctive mechanisms of resistance (e.g., G1407: PBP1 with L421, PBP2 pattern XXXIV, GyrA with S91F and D95G, ParC with S87R, PorB with G120K and A121N, *mtrR* promoter with A deletion).

**Conclusions:**

The prevalence of penicillin- ciprofloxacin- and tetracycline-resistant *Ng* has reached dramatic levels, whereas cefixime and ceftriaxone show MICs that tend to increase during time. International MDR clones less susceptible to cephalosporins are rapidly emerging indicating that the era of untreatable gonococcal infections is close.

## Background

*Neisseria gonorrhoeae* (*Ng*) is the etiologic agent of the second most common sexually transmitted infection (STI) globally [1]. The number of gonococcal infections is rapidly increasing, especially because subjects are often asymptomatic. This condition contributes to the spread of the pathogen, but also to the silent progression of the infection to more serious clinical conditions as the pelvic inflammatory diseases. Therefore, along with the implementation of effective preventive strategies, the administration of adequate antibiotic treatments can contribute to the control of infections [2,3].

Unfortunately, *Ng* has a remarkable ability to develop resistance to all of the clinically implemented antibiotics [4]. In brief, resistance to ciprofloxacin (CIP) is usually due to amino acid substitutions in GyrA and ParC [5,6]. Azithromycin (AZT) can become inactive due to mutations in the four copies of the 23S rRNA, production of methylase enzymes encoded by acquired genes (e.g., *ermB/F*), or substitutions in the L4 and L22 ribosomal proteins [5,7]. Tetracycline (TET) is usually ineffective because of *tet*(M) gene acquisition [3,5]. Resistance to penicillin (PEN) is due to production of acquired TEM-1-like β-lactamases or alterations of the penicillin binding proteins (PBPs; e.g., amino acid substitutions for PBP1 and PBP2) [3,4,8]. Notably, the over-expression of the MtrCDE efflux pump due to substitutions in its repressor MtrR or deletions/insertions in the *mtr* promoter region may reduce susceptibility to all of the above mentioned drugs [5,9], whereas substitutions in the PorB outer membrane porin may affect PEN and TET [10].

However, the most threatening global concern is represented by the recent emergence of *Ng* isolates resistant to cefixime (CFX) and, even more importantly, to ceftriaxone (CRO) [11-15]. Reduced susceptibility to these cephalosporins can be due to a mixture of the mechanisms involved in penicillin resistance, but full resistance necessitates specific mosaic structures of the PBP2 that are caused by recombination events occurring between gonococcus and other commensal *Neisseria* species [4,16,17].

Taking into account these clinical problems, and based on national and international guidelines [2,18], many countries have recently established active surveillance programs to monitor the evolution of antimicrobial susceptibility for *Ng* isolates [3,19-22], and the spread of international clones (e.g., sequence types ST1407, ST2992, and ST225) that are usually more resistant [8,13,19,23,24]. Clinical treatment and public health strategies for controlling gonococcal STI clearly benefit from the combined availability of these data [2,3]. However, we should note that there is a need of this compulsory information for our country. Thus, in the present work, we investigated the phenotypic and molecular characteristics of *Ng* isolates detected in Switzerland during a 15-year period.

## Methods

### Clinical isolates

We analyzed the *Ng* isolates collected by the Laboratory of Clinical Microbiology of the University of Bern (Switzerland) during a 15-year period and isolated implementing standard microbiological methodologies [25]. The characteristics of isolates detected during 1998–2001 (n = 26) were compared to those of gonococci detected during 2009–2012 (n = 34). Strains had been stored at -80°C over the time in glycerol stock tubes (Almedica AG). For the present study, isolates were plated on GC agar (bioMérieux) and species identification was confirmed by using the MALDI-TOF MS (Bruker Daltonik).

### Antimicrobial susceptibility tests (ASTs)

Minimum inhibitory concentrations (MICs) for PEN, CFX, CRO, CIP, AZT, TET, spectinomycin (SPE) and gentamicin (GEN) were obtained using the Etest method on GC agar plates. Results were categorized according to the 2013 European Committee on Antimicrobial Susceptibility Testing (EUCAST) criteria [26]. Production of β-lactamases was evaluated by implementing the cefinase disk (Oxoid) and also mixing 5 μL of β-lactamase(s) extract with 50 μL of nitrocefin [100 μM] for 1 hour. *N. gonorrhoeae* ATCC 49226 and *Staphylococcus aureus* ATCC 29213 were used as controls.

### Analysis of clonality

Sequence type (ST) designation was achieved by analyzing both *por* and *tbpB* genes according to the *Neisseria gonorrhoeae* Multi-Antigen Sequence Typing methodology (NG-MAST; http://www.ng-mast.net/) [27]. STs were clustered in genogroups (e.g., G1407, G2992, and G225) whenever possible, as previously done [23].

### Molecular characterization of drug resistance genes

Genomic extraction was performed using the QIAmp DNA mini kit (QIAGEN). The following antibiotic resistance genes (and their encoded proteins) were analyzed as previously described [5]: *ponA* (PBP1), *penA* (PBP2), *bla*_TEM_ (β-lactamases), *gyrA* and *parC* (quinolone resistance determining region), *penB* (PorB porin), *mtrR and mtrR* promoter (MtrCDE efflux pump), *rplD* and *rplV* (ribosomal protein L4 and L22, respectively), the four 23S rRNA gene copies, *mefA* (efflux-mediated resistance mechanism), *ermA/B/C/F* (methylase enzymes), and *tet*(M) (ribosomal protection protein). DNA sequencing was performed in service by Eurofins (Ebersberg, Germany) and chromatogram traces were analyzed using DNAStar (Lasergene). Results for all of the above genes were analyzed by searching for homologies with BLAST (http://blast.ncbi.nlm.nih.gov/Blast.cgi) or by comparison with the following deposited wild-type alleles: *ponA*, *penB*, *mtrR and mtrR* promoter, *gyrA*, *parC*, *rplD*, and *rplV* (GenBank: AE004969); *penA* (GenBank: M32091); *bla*_TEM-1_ (GenBank: AJPQ01000032); and 23S rRNA (GenBank: CP001050).

## Results

### Clinical isolates

*Ng* isolates collected during 1998–2001 were from 18 males (median age 26.5; range 0–37 years) and 8 females (median age 26.0; range 16–40 years), whereas those collected during 2009–2012 were from 30 males (median age 33.0; range 21–56 years) and 4 females (median age 25.0; range 23–49 years). In 1998–2001, samples from males were urethral (n = 17) and ocular swabs (n = 1), whereas those from females were cervical (n = 4), vaginal (n = 2), and wound swabs (n = 1), or sterile fluids (n = 1). With regard to 2009–2012, samples from males were urethral (n = 29) and pharyngeal swabs (n = 1), whereas those from females were vaginal (n = 3) and abscess swabs (n = 1).

### Antimicrobial susceptibility

**Figure 1:**
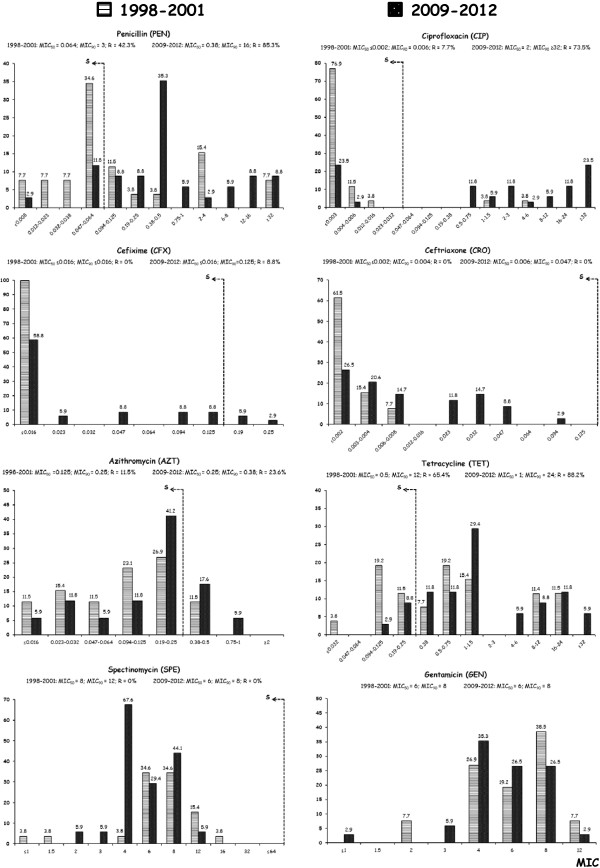
**MIC values distribution for the eight antibiotics tested against the*Ng *isolates detected during the two different periods (1998–2001*versus*2009–2012).** “S”, indicates the cut-off of susceptibility, whereas “R” indicates the isolates intermediate or resistant according to the EUCAST criteria [26]. The results clearly show that the MICs for cephalosporins increased over time (i.e., CRO: from MIC_90_ of 0.004 mg/L to 0.047 mg/L; CFX: from MIC_90_ ≤ 0.016 to 0.125 mg/L with also 8.8% of the isolates resistant) and that the prevalence of PEN-, CIP-, and TET-resistant isolates reached dramatic levels.

The MIC distributions for the eight antibiotics tested against *Ng* isolates detected during the two different periods are shown in Figure 1.

During the 15 years, gonococcal isolates showed a remarkable increase in resistance to PEN (from 42.3% to 85.3%) and CIP (from 7.7% to 73.5%), but also a marked rise in resistance to AZT (from 11.5% to 23.6%) and TET (from 65.4% to 88.2%). With regard to cephalosporins, we noted a rise in the MIC values for both CFX (from MIC_90_ ≤ 0.016 mg/L to MIC_90_ of 0.125 mg/L, with 8.8% of resistant isolates) and CRO (from MIC_90_ of 0.004 mg/L to MIC_90_ of 0.047 mg/L). Only SPE (all 60 isolates were susceptible) and GEN did not show increased MICs (Figure 1).

As shown in Table 1, the prevalence of multidrug-resistant (MDR) isolates (i.e., those resistant to ≥ 3 antibiotic classes [4]) increased from 7.7% in 1998–2001 to 70.6% in 2009–2012. We also noted that the isolates simultaneously resistant to four antibiotics augmented from 3.8% in 1998–2001 to 26.5% in 2009–2012, whereas those resistant to five antimicrobials (PEN, CIP, AZT, TET, and CFX) reached 5.9% in 2009–2012.

**Table 1:** **Patterns of antimicrobial and associated resistances of the *N. gonorrhoeae *** **isolates detected in Switzerland during the study period**^**a**^

**Antibiotics**^**b**^	**No. and (%) of isolates**	
	**1998-2001 (n = 26)**	**2009-2012 (n = 34)**
Resistant to ≥ 2 antibiotics:	14 (53.8)	31 (91.2)
-PEN and CIP	2 (7.7)	25 (73.5)
-PEN and AZT	3 (11.5)	8 (23.5)
-PEN and TET	14 (53.8)	28 (82.4)
-CIP and TET	2 (7.7)	23 (67.6)
-CIP and AZT	1 (3.8)	7 (20.6)
-AZT and TET	3 (11.5)	8 (23.5)
Resistant to ≥ 3 antibiotics (i.e., MDR isolates):	2 (7.7)	24 (70.6)
-PEN, CIP, and AZT	1 (3.8)	7 (20.6)
-PEN, CIP, and TET	2 (7.7)	23 (67.6)
-PEN, AZT, and TET	3 (11.5)	8 (23.5)
-CIP, AZT, and TET	1 (3.8)	7 (20.6)
Resistant to ≥ 4 antibiotics:	1 (3.8)	9 (26.5)
-PEN, CIP, AZT, and TET	1 (3.8)	7 (20.6)
-PEN, CIP, TET, and CFX	0 (0.0)	2 (5.9)
Resistant to 5 antibiotics (PEN, CIP, AZT, TET, and CFX)	0 (0.0)	2 (5.9)

**Note.** PEN, penicillin; CIP, ciprofloxacin; AZT, azithromycin; TET, tetracycline; CFX, cefixime.

^**a**^All isolates were susceptible to ceftriaxone (CRO) and spectinomycin (SPE).

^**b**^“Resistant” includes intermediate and resistant according to the EUCAST criteria [26].

### Clonality

**Figure 2:**
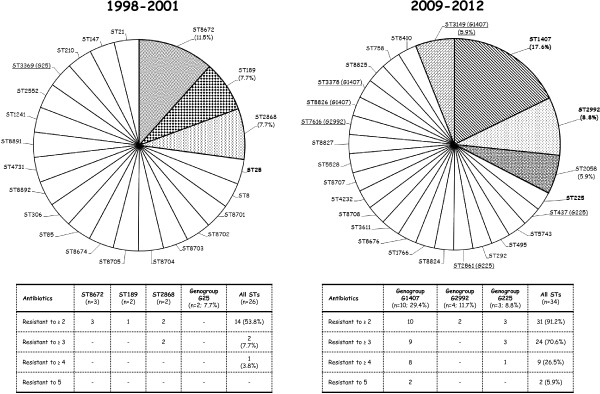
**Sequencing types (STs) of the*Ng *isolates detected during the two different periods (1998–2001 and 2009–2012).** STs with more than one isolate are indicated in colors and with their percentages. Hyperepidemic clones are indicated in bold and STs included in the same genogroup (G) are underlined. The results show that international lineages reported in other countries (G1407, G2992, and G225) are also emerging and spreading in Switzerland. With the exception of two isolates of G25, these STs/Gs were not present during the previous decade. Notably, most of the hyperepidemic clones were MDR (i.e., resistant to ≥ 3 antibiotics).

Sequencing types (STs) of *Ng* isolates detected during 1998–2001 and 2009*–*2012 are summarized in Figure 2.

In 1998–2001, only the international genogroup G25 was identified (one ST25 in 1998 and one ST3369 in 2001). In contrast, during 2009–2012 the international lineages G1407 (29.4%), G2992 (11.7%), and G225 (8.8%) were detected. Most of these hyperepidemic genogroups were MDR (e.g., 9/10 of the isolates of G1407 were resistant to ≥ 3 antibiotics). Moreover, *Ng* of G1407 possessed the highest MICs for CFX and CRO, with MIC_50/90_ of 0.125/0.19 mg/L and MIC_50/90_ of 0.032/0.047 mg/L, respectively (data not fully shown; see also Table 2).

**Table 2:** **Phenotypic and molecular characteristics (clonality and drug resistance genes) of 26** ***N. gonorrhoeae *** **isolates detected during the period 2010–2012 in Switzerland**

**Isolate**	**Year**	**ST (Genogroup)**	**PP**^**a**^	**Minimum inhibitory concentration, mg/L**^**b**^								**PBP1 (*ponA*)**	**PBP2 (*penA*)**^**c**^	**TEM**	**GyrA**	**ParC**	**PorB (*penB*)**	**MtrR**	***mtrR*** **promoter**	**L4 (*rplD*)**	**L22 (*rplV*)**	**23S rRNA (4 copies)**	***ermA/B/C/F and mefA***	***tet(M)***
				**PEN**	**CFX**	**CRO**	**CIP**	**AZT**	**TET**	**SPE**	**GEN**													
1820606	2010	495	+	6	≤0.016	0.006	0.006	0.38	96	12	8	L421P	XII	TEM-1	WT	WT	G120K, A121D	A39T, L47P	WT	WT	WT	All WT	-	+
1865296	2010	292	-	0.25	≤0.016	0.006	2	0.25	0.5	6	4	L421P	V	-	S91F, D95G	S87R	A121S	WT	A deletion	WT	WT	All WT	-	-
1872778	2010	2861 (G225)	-	0.38	0.047	0.032	>32	0.25	0.75	4	4	L421P	XII	-	S91F, D95G	S87R	G120K, A121D	WT	A deletion	WT	WT	All WT	-	-
1832086	2010	1407 (G1407)	-	0.5	0.19	0.047	6	0.25	1.5	8	6	L421P	XXXIV	-	S91F, D95G	S87R	G120K, A121N	WT	A deletion	WT	WT	All WT	-	-
1859198	2010	225 (G225)	-	0.5	0.047	0.032	>32	0.38	1.5	8	8	L421P	XII	-	S91F, D95G	S87R	G120K, A121D	WT	A deletion	WT	WT	All WT	-	-
1910312	2010	2992 (G2992)	-	0.094	≤0.016	0.003	≤0.002	0.25	0.38	6	4	WT	II	-	WT	WT	WT	A39T, R44H	WT	WT ^d^	WT	All WT	-	-
1869994	2010	1766	+	6	≤0.016	0.006	0.75	≤0.016	6	6	4	L421P	IIg	TEM-1	S91F, D95A	S87N	G120D	WT	A deletion	WT	WT	All WT	-	+
1912168	2010	8676	+	32	≤0.016	≤0.002	3	0.032	8	4	4	L421P	II	TEM-1	S91F, D95G	S87R	G120K, A121D	WT	WT	WT	WT	All WT	-	+
1922603	2011	3149 (G1407)	-	0.38	0.19	0.047	16	0.25	1.5	8	6	L421P	XXXIV	-	S91F, D95G	S87R	G120K, A121N	WT	A deletion	WT	WT	All WT	-	-
1996003	2011	3611	+	12	≤0.016	0.008	>32	0.125	96	8	4	L421P	XIX	TEM-1	S91F, D95A	S87N, E91K	G120K, A121G	A39T	WT	WT	WT	All WT	-	+
2017570	2011	1407 (G1407)	-	0.38	0.125	0.023	12	0.38	1	8	6	L421P	XXXIV	-	S91F, D95G	S87R	G120K, A121N	WT	A deletion	WT	WT	All WT	-	-
2004277	2011	8708	-	0.125	≤0.016	≤0.002	≤0.002	0.25	0.5	8	4	WT	II	-	WT	WT	WT	G45D	WT	WT	WT	All WT	-	-
1973517	2011	4232	-	0.19	≤0.016	0.003	≤0.002	0.19	16	8	6	WT	II	-	WT	WT	A121S	G45D	WT	WT ^d^	WT	All WT	-	+
1929711	2011	2992 (G2992)	-	0.064	≤0.016	0.003	≤0.002	0.25	0.38	8	6	WT	II	-	WT	WT	WT	A39T, R44H	WT	WT ^d^	WT	All WT	-	-
1944251	2011	2992 (G2992)	-	0.047	≤0.016	0.003	≤0.002	0.25	0.25	6	4	WT	II	-	WT	WT	WT	A39T, R44H	WT	WT ^d^	WT	All WT	-	-
1954615	2011	8707	+	12	≤0.016	0.004	1.5	0.064	24	2	4	L421P	XIX	TEM-1	S91F, D95A	S87R	A121S	A39T	WT	WT	WT	All WT	-	+
1946253	2011	1407 (G1407)	-	0.38	0.125	0.032	32	0.75	1.5	8	6	L421P	XXXIV	-	S91F, D95G	S87R	G120K, A121N	WT	A deletion	WT	WT	All WT	-	-
2036196	2012	5528	+	>32	≤0.016	0.004	12	0.25	64	8	4	WT	II	TEM-135	S91F, D95G	D86N	G120K, A121D	WT	WT	WT	WT	All WT	-	+
2086772	2012	8827	+	3	≤0.016	≤0.002	2	0.023	16	6	6	L421P	XIX	TEM-1	S91F, D95A	S87N, E91K	G120K, A121D	A39T	WT	WT	WT	All WT	-	+
2096513	2012	7616	-	0.047	≤0.016	0.002	≤0.002	0.25	0.38	6	8	WT	II	-	WT	WT	WT	A39T, R44H	WT	WT ^d^	WT	All WT	-	-
2112655	2012	8826	-	0.38	0.094	0.023	24	0.125	0.25	3	6	L421P	XXXIV	-	S91F, D95G	S87R	G120K, A121N	WT	A deletion	WT	WT	All WT	-	-
2119347	2012	3378	-	0.38	0.094	0.032	16	0.5	1	8	12	L421P	XXXIV	-	S91F, D95G	S87R	G120K, A121N	WT	A deletion	WT	WT	All WT	-	-
2121127	2012	8825	-	0.125	≤0.016	0.003	0.75	≤0.016	0.25	2	1	L421P	II	-	S91F, A92S, D95N	E91G	G120K, A121D	A39T	WT	WT	WT	All WT	-	-
2129562	2012	437 (G225)	-	0.25	0.023	0.023	24	0.19	0.5	3	8	L421P	V	-	S91F, D95G	S87R	G120K, A121D	WT	A deletion	WT	WT	All WT	-	-
2131245	2012	8410	+	16	0.023	0.006	3	0.064	8	8	6	WT	XXI-like	TEM-135	S91F, D95A	S87R	A121G	A39T	WT	WT	WT	All WT	-	+
2132307	2012	3149 (G1407)	-	0.38	0.094	0.023	32	0.38	1	8	8	L421P	XXXIV	-	S91F, D95G	S87R	G120K, A121N	WT	A deletion	WT	WT	All WT	-	-

**Note.** WT, wild-type; +, positive; -, negative.

^**a**^Penicillinase producers according to the cefinase disk test (Oxoid) and nitrocefin test.

^**b**^Abbreviations and MIC interpretation according to the EUCAST criteria [26]: penicillin (PEN; sensible, S ≤ 0.06 mg/L); ceftriaxone (CRO; S ≤ 0.125 mg/L); cefixime (CFX; S ≤ 0.125 mg/L); ciprofloxacin (CIP; S ≤ 0.03 mg/L); azithromycin (AZT; S ≤0.25 mg/L); tetracycline (TET; S ≤ 0.25 mg/L); spectinomycin (SPE; S ≤ 64 mg/L); gentamicin (GEN; criteria not available).

^**c**^The characteristics of the PBP2 patterns II, V, XII, and XIX were described by Whiley DM *et al*. [16], those of pattern XXXIV by Hess D *et al*. [28], and pattern IIg by Takahashi *et al*. [29]. Pattern XXI-like does not have -574N and A575V as in the XXI [16].

^**d**^The L4 of these *Ng* isolates possessed three substitutions (i.e., V125A, A147G, R157Q) that have no recognized impact on the macrolides resistance.

### Drug resistance genes

The specific phenotypic and molecular characteristics of *Ng* detected during 2010–2012 (n = 26) are presented in Table 2.

Isolates with reduced susceptibility to antibiotics possessed the following specific antibiotic resistance traits: PBP1 (substitution L421P), PBP2 (mosaics II, V, XII, XIX, or XXXIV), TEM-1-like β-lactamases, GyrA (substitutions S91F, D95G, and D95A), ParC (substitutions S87R and E91G), PorB (substitutions G120K and A121D/N/G), MtrR (substitutions A39T, R44H, G45D, L47P), *mtrR* promoter (A deletion), and the Tet(M) protective protein. *RplD*, *rplV* and 23S rRNA genes did not present mutations and *erm* or *mefA* genes were not detected.

Notably, *Ng* isolates belonging to G1407, G2992, and G225 constantly possessed specific and conserved antibiotic resistance patterns (e.g., all G1407 had PBP1 with L421P, PBP2 pattern XXXIV, GyrA with S91F and D95G, ParC with S87R, PorB with G120K and A121N, and the A deletion in the *mtrR* promoter region).

## Discussion

Programs monitoring the antibiotic susceptibility of *Ng* are essential to implement adequate empirical treatments [1,3,18,20]. Information about the underlying molecular mechanisms of resistance is equally important to design and evaluate new rapid diagnostic systems [30]. Moreover, the incidence of *Ng* hyperepidemic clones should be carefully monitored because these isolates have the potential to spread quickly and are showing increasing resistance to most of the clinically used antibiotics [13,19,23].

In 2008, Le Lin *et al*. described the emergence (2002) of *Ng* resistant to CIP in Geneva [31]. Very recently, Kovari *et al*. have analyzed the susceptibility to four antibiotics (CFX, CRO, CIP and PEN) of 320 isolates collected in 1990 and from 2000 to 2012 at the University of Zurich [32]. However, up to now surveys studying the molecular characteristics, especially the clonality, of *Ng* detected in Switzerland have not yet been performed. Therefore, the aim of the present work was to fill up the lack of contemporary data regarding gonococcus implementing the methodologies suggested by the acknowledged international institutions [19,20].

### Trend of antibiotic susceptibility

Consistently with the study of Kovari *et al*. [32], our phenotypic data indicate that the prevalence of *Ng* resistant to either PEN or CIP increased considerably during the last decade (>73%). However, in our study we have also observed that the occurrence of isolates simultaneously resistant to PEN, CIP, and TET has reached a dramatic level (68%). These figures clearly show that the use of these three antibiotics for the empirical treatment of gonococcal infections is no longer safe. This phenomenon is not new and has been observed in other countries. The extensive use of antibiotic treatments without performing the ASTs has generated not only an escalation of “treatment failures” [33-36], but also a positive selective pressure for specific international MDR clones (e.g., G1407 and G225) [3,4].

Fortunately, current *Ng* isolates spreading in Switzerland seem to remain susceptible to the standard therapeutic option CRO [32,37]. However, its MICs are increasing over time (“MIC creep”) as observed worldwide [3,4,20], and one should be aware that CRO-resistant isolates can emerge and spread [4,7,11,12,14,17]. This possible development could also be favored by the observed decreased susceptibility to AZT, an antibiotic used together with CRO to cover *Chlamydia trachomatis* co-infections and more recently also implemented to prevent treatment failures of gonococcal infections [33,37]. In this overall scenario, we noted that all *Ng* tested were susceptible to SPE, probably because the use of this antibiotic has been abandoned after observing outbreaks of resistant isolates in the 1980s [3,4]. SPE-resistant *Ng* are now rarely reported [20,21], and this antibiotic could be reconsidered - along with gentamicin - for the treatment of infections due to MDR isolates showing co-associated reduced susceptibility to CRO and AZT [4].

### Emergence of successful international STs and genogroups

The NG-MAST analysis pointed out that in the last few years the G1407, G2992, and G225 international genogroups have also reached Switzerland, now representing ~50% of the overall isolates. Most likely, these lineages were initially imported by infected people who had sexual intercourse abroad, but it is difficult to hypothesize which countries were most responsible for this phenomenon. In fact, G1407 is currently the most frequently detected genogroup worldwide (e.g., 23% of the isolates in Europe), whereas G225 and G2992 are second and third (e.g., 10% and 8% in Europe, respectively) [23]. For instance, ST1407 is first in rank in countries like Austria, Italy, the Netherlands, Portugal, Spain, Romania, Slovenia, United Kingdom, Japan and Canada; ST2992 is very frequent in Ireland and Norway; and ST225 is predominant in Malta and Denmark [19,21,24,38].

As also observed in this work, G1407 and G225 are constantly characterized by fully resistance to CIP and reduced susceptibility (at intermediate level) to PEN, AZT, and TET, whereas G2992 is usually pan-susceptible [3]. G1407 has also decreased susceptibility to CFX and, to a lesser extent, to CRO [4,21,28]. In this context, we note that most of the previously reported CFX or CRO treatment failures were due to *Ng* isolates of G1407 [4,11,12,39].

### Drug resistance genes

Due to the number and complexity of the mechanisms [5], we decided to analyze only the main molecular traits of resistance possessed by the *Ng* isolates found during 2010–2012 (Table 2).

Isolates of G1407 were slightly resistant to PEN because of L421P in PBP1, overexpression of MtrCDE efflux by one A deletion in the promoter, and reduced permeability of PorB (substitutions G120K and A121N). However, they also showed augmented MICs to CFX and CRO due to the presence of the mosaic XXXIV [4,8,21,28,36,38]. This PBP2 variant may predispose the clone to become highly-resistant to CRO [11,12]. Similarly, G2992 and G225 tested intermediate to PEN, but were fully susceptible to cephalosporins because of the presence of PBP2 mosaics (i.e., II, V, XII, and XIX) that have little effects against CFX and CRO [16]. These commonly reported PBP2 mosaics were also present in the fully cephalosporin-susceptible *Ng* belonging to sporadic STs.

Non-hyperepidemic isolates were frequently TEM-1 producers but two of them expressed the TEM-135, a TEM-1 variant (substitution M182T) previously reported only in Japan, Thailand, and China [8,40,41]. TEM-135 is a plasmid-mediated enzyme that does not hydrolyze CFX and CRO, but its emergence might represent an intermediate stage of the traditional TEM-1 penicillinase into an extended-spectrum β-lactamase (ESBL) that is able to confer high-level resistance to all β-lactams with the exception of carbapenems [4]. Since this was not a prospective clinical study, we are unable to provide information about the travel history of the two patients infected by TEM-135 producers.

None of the *Ng* tested possessed acquired genes (*erm*, *mefA*) or chromosomal mutations (23S rRNA, *rplD*, *rplV*) conferring high-level resistance to macrolides [5,7]. However, several isolates (including some of G1407 and G225) were non-susceptible to AZT (MICs of 0.38-0.75 mg/L). This phenomenon may be due to a feeble over-expression of the MtrCDE efflux induced by the A deletion in the *mtrR* promoter [5,9,42]. Finally, almost all CIP-resistant *Ng* possessed well-known substitutions in GyrA (amino acids 91 and 95) and ParC (amino acid 87). In particular, we highlight that the S91F replacement in GyrA was constantly observed, making it a good single target candidate for the molecular tests functional to categorize *Ng* as resistant or susceptible to quinolones [6]. The implementation of a rapid bedside molecular test for CIP resistance would allow treatment of infections due to susceptible isolates with this drug and could reduce the selective pressure on CRO in about 25% of gonorrhea in our country while still maintaining good clinical efficacy.

## Conclusions

This is the first study describing clonality and molecular mechanisms of resistance of gonococcal isolates detected in Switzerland. In this survey, we also analyzed the changes in characteristics of *Ng* strains detected in the same geographic area and during the last 15 years. We note that studies focusing on these epidemiological temporal trends are still limited but extremely important to comprehend the spread and the evolution of antibiotic resistance in *Ng*[3,20,22,32].

Overall, our data clearly indicate that the antibiotic susceptibility of contemporary *Ng* isolates is dramatically decreasing, mainly due to the spread of international MDR clones that also show reduced susceptibility to the standard therapeutic options CRO and AZT [37]. These specific clones (e.g., ST1407 and its genogroups) are predisposed to become more resistant representing the last evolutionary step of the pathogen before the era of extensively and pandrug-resistant isolates [4]. In this context, we emphasize the urgent need of: *i*) rapid molecular tests able to provide adequate information to select the most appropriate direct antibiotic treatment [30]; *ii*) novel antimicrobial families against molecular targets not yet affected by resistance mechanisms [4]; *iii*) increasing numbers of cultures to study and monitor clonality and resistance mechanisms of gonococcus [4].

One limitation of the present study is that the survey was performed in a single institution, was not prospective, and included a relative small number of *Ng* isolates. However, we believe that our results may be representative for the whole country because distances in Switzerland are very short and our institution is located in the middle of the territory. In support of this speculation, we note that our phenotypic data are consistent with those recently provided by the larger analysis performed in Zurich [32].

To provide more robust and systematic data that are essential for controlling the rapidly increasing number of gonococcal STI, we strongly believe that an adequate national surveillance monitoring program - supported by the state public institutions - should be established rapidly and before our health care systems dramatically face the attack of untreatable gonococcal isolates [4,17].

## Competing interests

The authors declare that they have no competing interests.

## Authors’ contributions

AE: study design, results interpretation, and writing manuscript; YNG: molecular characterization, species identification, phenotypic tests; RT: molecular characterization; LH: molecular characterization and phenotypic tests; SS: species identification, phenotypic tests; AL: molecular characterization and reviewing manuscript; CH: reviewing manuscript; HF: study design and reviewing manuscript. All authors read and approved the final manuscript.

## Pre-publication history

The pre-publication history for this paper can be accessed here:

http://www.biomedcentral.com/1471-2334/14/106/prepub
